# Identification of Simultaneous Occurrence of Amphibian Chytrid Fungi and Ranavirus in South Korea

**DOI:** 10.3390/ani15142132

**Published:** 2025-07-18

**Authors:** Ji-Eun Lee, Young Jin Park, Mun-Gyeong Kwon, Yun-Kyeong Oh, Min Sun Kim, Yuno Do

**Affiliations:** 1 Department of Biological Sciences, Kongju National University, Gongju 32588, Republic of Korea; jelee00@smail.kongju.ac.kr (J.-E.L.); jintall123@naver.com (Y.J.P.); 2 Aquatic Disease Control Division, National Fishery Products Quality Management Service (NFQS), Busan 49111, Republic of Korea; mgkwon@korea.kr (M.-G.K.); oskal@korea.kr (Y.-K.O.)

**Keywords:** amphibian decline, *Batrachochytrium dendrobatidis*, disease epidemiology, life history, sexual selection, pathogen co-occurrence, prevalence

## Abstract

Emerging infectious diseases such as chytridiomycosis and ranavirosis, caused by *Batrachochytrium dendrobatidis* (*Bd*) and ranavirus (RV), respectively, are major contributors to global amphibian declines. Despite their significance, comprehensive data on the spatial epidemiology of these pathogens in South Korea remain limited. This study aimed to assess the nationwide co-occurrence and prevalence of *Bd* and RV across four anuran species in five administrative regions. Infection rates were analyzed in relation to host species, sex, and life history stage. Results indicated distinct prevalence patterns driven by ecological traits. *Bd* was predominantly detected in mountainous and coastal habitats, whereas RV was more common in flat inland areas. Both pathogens exhibited peak occurrence in central regions, likely reflecting seasonal transmission dynamics rather than stable endemic hotspots. The observed spatial heterogeneity appears to be influenced by pathogen-specific thermal tolerance and host ecology. These findings underscore the importance of understanding host–pathogen–environment interactions for effective disease surveillance and management. Continuous monitoring and integrative ecological approaches are essential to mitigate pathogen-induced biodiversity loss and to inform amphibian conservation strategies in East Asia.

## Simple Summary

Amphibian population declines are driven by emerging infectious diseases, particularly chytridiomycosis and ranavirosis, caused by the chytrid fungus and ranavirus. This study assessed the nationwide distribution of these pathogens in South Korea by examining infection prevalence across species, sex, and life stages. Frogs from multiple regions were sampled, revealing that the chytrid fungus was prevalent in mountainous and coastal areas, while ranavirus occurred mainly in lowland regions. Both pathogens co-occurred most frequently in central Korea, likely due to seasonal dynamics rather than true hotspots. These patterns reflect species-specific ecological traits and pathogen thermal preferences. Understanding these dynamics is crucial for disease management, and long-term monitoring is recommended to support amphibian conservation.

## 1. Introduction

Amphibians are currently experiencing one of the most severe extinction events in vertebrate history [1]. Particularly, large-scale pandemics such as those caused by *Batrachochytrium dendrobatidis* (*Bd*), an amphibian chytrid fungus, and ranavirus (RV), a member of the Iridoviridae family, have been cited as one of the primary causes of amphibian extinctions [1]. Therefore, many researchers have been studying global population declines, infection mechanisms, lineage origins, and disease dynamics following major infectious diseases that threaten amphibian populations [2,3,4,5,6]. *Bd* infects the keratinized epidermis of amphibians, causing chytridiomycosis, a disease that leads to epidermal hyperkeratosis. This condition disrupts essential ion transport across the skin, often resulting in osmotic imbalance, cardiac arrest, and death [7]. In contrast, RV primarily targets internal organs such as the liver and kidneys, causing hemorrhage, necrosis, and systemic organ failure [8]. Both pathogens can induce mass mortality events and have been implicated in rapid population declines across diverse amphibian communities.

To conserve amphibians, it is essential to predict and manage pathogen epidemiology [9]. The most basic task is to understand the distribution and prevalence of diseases at the regional scale [10]. In the case of *Bd*, a previous nationwide field investigation of South Korea reported distinct differences in the prevalence of species and sites, with higher infection rates documented in amphibians associated with humid or aquatic habitats [11]. Similarly, a recent nationwide field investigation of RV in the Korean Peninsula clearly showed that the prevalence differed by species and region [12]. Despite the likelihood that *Bd* and RV overlap geographically, no study has yet carried out a simultaneous, country-wide assessment of both pathogens in South Korea. Such work must also account for interspecific differences in prevalence, because host-specific variation can shape broader patterns of pathogen distribution and disease risk. Interspecific differences in pathogen prevalence are a global concern and are influenced by the physiological, genetic, and ecological traits of amphibian hosts. For example, the ability to tolerate environmental tolerances of host species, especially thermal variation, can affect their susceptibility to *Bd* and RV [13,14]. For species restricted to high elevations or cold climates, limited habitat options can intensify the effects of pathogens whose growth is closely tied to temperature [15]. Host phylogeny likewise shapes infection risk, since closely related species often share similar levels of susceptibility. For instance, variation in MHC class II alleles, as well as differences in skin microbial communities associated with host genetics, have been shown to contribute significantly to resistance against *Bd* [16]. These immune-related traits are often conserved within certain lineages, which may explain taxon-specific patterns of vulnerability [17]. Ecological characteristics also play a significant role and may interact with physiological and genetic factors. *Bd* and RV are waterborne pathogens and primarily spread through aquatic environments [18,19]. Therefore, seasonal variation in water availability, along with species-specific breeding, hibernation, and movement behaviors, plays a critical role in shaping the risk of pathogen exposure [19,20]. However, recent studies have shown that these pathogens can also persist in moist substrates such as wet soil, leaf litter, or sediment, and may be transmitted indirectly through invertebrate vectors, adding further complexity to their transmission dynamics [2,21,22]. Additionally, close physical contact among individuals during the breeding season further increases opportunities for transmission [23]. Because breeding periods are closely tied to seasonal cycles, the timing and duration of reproductive activity can significantly influence exposure risk and shape pathogen transmission patterns across different species.

In addition to prevalence differences by species, infections vary greatly depending on the life cycle stage and sex. Unlike other vertebrates, amphibians have a complex life cycle and show various responses [24]. The prevalence and epidemiology of the pathogens are also distinct [25]. In general, RV is known to have a more severe effect on the larval stage of the amphibian, which is often an aquatic life stage, than on the post-metamorphosis life cycle [26,27]. Moreover, the mortality rates due to RV infection vary even within the development stages of the larval frogs (tadpoles) [28]. In contrast, *Bd* has a nonlethal effect during the tadpole stage, but has a virulent effect on amphibians after metamorphosis [29]. In particular, *Bd* is attracted to thyroid hormones produced during metamorphosis, which increases its colonization and pathogenicity in the host [30]. These results highlight the importance of studying the prevalence across life history stages and species. In addition, sex differences may affect infection owing to reproductive behavior and energy distribution [31]. While female frogs expend a great deal of energy during the egg-laying process, male frogs experience continuous energy consumption and risk through territorial defense and vocalization behaviors during the breeding season [32,33]. These ecological and behavioral characteristics must be studied in detail as they may influence differences in pathogen exposure.

This study aimed to (a) understand the lineages and distribution of *Bd* and RV, two major pathogens affecting amphibians in South Korea, (b) provide a comprehensive report on the national distribution of *Bd* and RV in multiple species, as well as the prevalence of *Bd* and RV in each species in each region, (c) identify areas where the two pathogens were co-distributed, and (d) inspect for differences in prevalence according to sex and life history.

## 2. Materials and Methods

### 2.1. Site Selections

**Figure 1:**
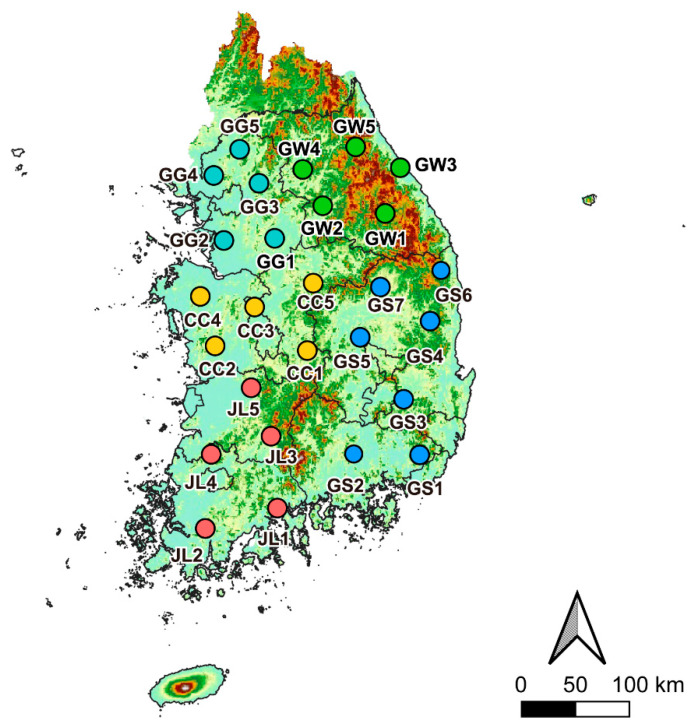
Each investigation site in the five administrative regions selected in this study: five sites in Gyeonggi-do (GG1–GG5, light blue color), five sites in Gangwon-do (GW1–GW5, green), five sites in Chungcheong-do (CC1–CC5, yellow), five sites in Jeolla-do (JL1–JL5, red), and seven sites in Gyeongsang-do (GS1–GS5, blue).

We collected frogs from five provinces among the 17 first-level administrative regions in South Korea to support future disease management strategies based on administrative boundaries. A total of 27 paddy fields were selected as field sites across the five provinces: five sites in Gyeonggi-do, five in Gangwon-do, five in Chungcheong-do, five in Jeolla-do, and seven in Gyeongsang-do (Figure 1). In South Korea, rice paddies function as semi-natural wetland agroecosystems that follow a distinct hydrological rhythm dictated by cultivation. From roughly May to September, farmers maintain a shallow water layer over the basins; once the harvest is complete, the fields are drained and either left fallow or prepared for winter crops. Earthen levees separate individual plots, while the surrounding landscape is typically a patchwork of secondary vegetation and other lowland farmland. During the flooded months, the combination of high humidity, stable water temperatures, and abundant invertebrate prey offers amphibians a reliable setting for courtship, egg-laying, and larval development, effectively turning paddy fields into seasonal breeding refuges. The sites we surveyed account for roughly 29% of all administrative regions nationwide.

We determined that late spring to early summer was the most appropriate period for monitoring the two pathogens. *Bd* is known to thrive under cooler conditions, particularly at temperatures between 17 and 25 °C [34], whereas RV exhibits elevated viral titers under warmer conditions, typically around 28–30 °C [35]. Considering these optimal thermal conditions of the two pathogens, we concluded that this intermediate period increases the likelihood of their coexistence, thereby providing an optimal window for simultaneous detection. Therefore, individuals were collected from 27 paddy fields between late May and mid-June of 2024 (Table 1). Sampling was conducted independently by two teams, each consisting of three members, with 2–3 sites sampled per day. Within each team, one person was responsible for capturing frogs, while the remaining two handled swabbing and labeling. These roles remained fixed throughout the entire sampling period.

Four common anuran species were selected based on their wide distribution across the country and their representativeness of different ecological traits. These included *Dryophytes japonicus*, *Pelophylax nigromaculatus*, *Glandirana rugosa*, and *Bombina orientalis*. All four species are listed as Least Concern on the IUCN Red List and are not designated as protected species in Korea.

### 2.2. Species Selection

We focused on four anuran species with high densities during this period because of their breeding activities. Despite the selection of sites where all four species were likely to occur, we were unable to collect all four species from all sites. *D. japonicus* was collected from all 27 sites in the five regions, and *P. nigromaculatus* was collected from 25 sites in the five regions. *G. rugosa* and *B. orientalis* were difficult to collect together and were only sampled from a few sites. *G. rugosa* was collected from six sites in four administrative regions, excluding Gangwon-do, whereas *B. orientalis* was collected from five sites in Gangwon-do and Gyeongsang-do.

Although these species share similar breeding periods, their ecological characteristics differ. For instance, *D. japonicus*, an arboreal frog, utilizes temporary wetlands, such as paddy fields, during the breeding season and transitions to terrestrial habitats, such as trees or grass, during the non-breeding season [36]. Similarly, *B. orientalis* tends to use temporary wetlands, such as paddy fields, only during the breeding season, and during the non-breeding season, it migrates to valleys or water edges, where it lives as a semi-aquatic frog [37]. Consequently, we were only able to collect reproductively mature Japanese tree frogs and fire-bellied toads that participated in breeding activities in the paddy fields.

However, we were able to collect various stages of *P. nigromaculatus* and *G. rugosa* in the paddy fields. *P. nigromaculatus* is a semi-aquatic frog that exhibits philopatry at breeding sites, allowing multiple life stages to be observed in paddy fields [38]. *G. rugosa* has a relatively late breeding period compared to the other three species and can hibernate in its tadpole stage as a semi-aquatic amphibian [39]. Because of these traits, we were able to collect juvenile, subadult, and reproductively mature adult frogs of *P. nigromaculatus* and *G. rugosa* from the paddy fields.

The collected frogs were used for sex and development stage identification. Adult male frogs were identified by the presence of nuptial bumps on their forelimbs during the breeding season. Individuals were considered female if they were larger than males, had relatively thin forelimbs, and lacked a vocal sac and nuptial bump. Non-adult frogs were categorized into juvenile and subadult frogs. Frogs that had metamorphosed in the previous year were considered juvenile frogs, and individuals that had metamorphosed for two years or more were considered subadult frogs. In *P. nigromaculatus* and *G. rugosa*, the juvenile and subadult frogs were separated by size. In the case of *G. rugosa*, we were unable to identify individuals in the metamorphic or froglet stages, although fresh froglets were present in the spring.

### 2.3. Sample Collection

In the field, the frogs were collected by hand using sterile gloves, and a new pair of sterile gloves was used each time a frog was collected to prevent cross-contamination by microorganisms. After capture, the frogs were immediately sampled for *Bd* infection in the field. The frogs were rubbed on their ventral side, inner leg, toes, and fingers five times each using sterile Isohelix DNA Buccal Swabs (SK-2S, Isohelix™, Harrietsham, UK) [40]. The swabs were transferred to sterile 2 mL microcentrifuge tubes, and only the heads of the swabs were stored after removing the sticks through the break point. The swabs were stored at 4 °C in the field and then transferred to the Animal Laboratory of Kongju National University and stored at −20 °C until DNA extraction. Extraction of gDNA was performed using PrepMan™ Ultra Sample Preparation Reagent (Applied Biosystems™, Waltham, MA, USA) according to the manufacturer’s protocol [40].

After swabbing, the frogs were placed individually in sterilized plastic bags and labeled on site, then transferred to the laboratory. The frogs were humanely euthanized using 5 g/L MS-222 (tricaine methane sulfonate) solution for 5 min without stress and dissected to collect the liver and kidneys for detection of RV infection [41,42,43]. We extracted gDNA from the organs using a Qiagen Blood & Tissue Kit (Qiagen, Hilden, Germany) and stored it at −20 °C until RV detection was performed [44]. All animal maintenance and experimental procedures received approval from the Experimental Animal Ethics Committee of Kongju National University (Approval No. KNU_2024–01).

### 2.4. Molecular Detection of Bd and RV

The gDNA extracted from swabs was used to detect *Bd* infection using TaqMan real-time PCR. Referring to the previous study, the ITS1-5.8S-2 region of 140 bp in size was amplified from the obtained gDNA using primers ITS1-3 Chytr (5′-CCTTGATATAATACAGTGTGCCATATGTC-3′), Chytr 5.8S (5′-6FAM-CGAGTCGAACAAAAT-MGBNFQ-3′), and Chytr MGB2 probe (5′-FAM-CGAGTCGAACAAAAT-MGBNFQ-3′) [40,45]. TaqMan PCR was performed in triplicate on all samples and the positive and negative controls (distilled water) using Bio-Rad CFX Duet (Bio-Rad Laboratories, Hercules, CA, USA) in a total volume of 20 µL using 10 µL SsoAdvanced Universal Probes Supermix 2× from Bio-Rad. The positive control consisted of *Bd* DNA extracted from a wild-caught *Bombina orientalis* that was confirmed to be *Bd*-positive by nested PCR targeting the ITS-1/5.8S rRNA region using skin microbial DNA [46]. The amplified product was verified by Sanger sequencing, and the resulting sequence was aligned with reference *Bd* sequences in GenBank (accession no. JQ582891.1) to confirm species specificity [47]. Based on the verified sequence, *Bd*-specific oligonucleotides with no significant similarity to non-*Bd* sequences were synthesized and used as the positive control. The amplification process was performed in three steps: pre-heating at 50 °C for 2 min, then 95 °C for 10 min; 45 cycles at 95 °C for 15 s and 60 °C for 1 min, and extension reaction at 40 °C for 30 s. For relative quantification, the positive and negative controls (distilled water) were amplified together with the samples. The quantification cycle (Cq) value for each sample was calculated using CFX Maestro software (version 2.3) provided by Bio-Rad, and the frogs positive or negative for *Bd* were determined. According to the World Organization for Animal Health (WOAH), a Cq value of 39 or lower is considered positive [48].

Similarly, we performed TaqMan real-time PCR using gDNA from the liver tissue to detect RV infection. The 97 bp major capsid protein (MCP) was amplified using RanaF1 (5′-CCAGCCTGGTGTACGAAAACA-3′), RanaR1 (5′-TATGCCACCTCCATCCCAGT-3′) primers, and RanaP1 probe (5′-6FAM-TGGGAGTCGAGTACTAC-MGBNFQ-3′) according to methods described in a previous study [49]. For relative quantification, the positive and negative controls (distilled water) were amplified together with the samples. The positive control employed in this study was synthesized based on the reference nucleotide sequence of Frog Virus 3 (Gene ID: 2947809) retrieved from the NCBI database [50]. The amplification was performed using a Light Cycler 96 instrument (Roche, Basel, Switzerland) with pre-heating at 50 °C for 2 min, and then 95 °C for 10 min; The amplification reaction was performed under the conditions of 95 °C for 15 s, 50 cycles at 60 °C for 30 s, and the extension reaction was performed at 40 °C for 30 s. After amplification, LightCycler 96 software (version 4.1) provided by Roche was used to calculate the Cq value for each sample along with the amplification curve, and the frogs’ positive or negative status for RV was determined. RV positivity was determined to be positive when the Cq value was ≤35 [12,51].

### 2.5. Lineage Verification of Bd and RV

To identify the lineages of *Bd*, nested PCR was performed on the samples that were positive. Using 12.5 µL Takara (R051A, Tokyo, Japan), a 500 bp region of 18S-28S was amplified using the *Bd*18SF1 (5′-TTTGTACACACCGCCCGTCGC-3′) and *Bd*28SR1 (5′- ATATGCTTAAGTTCAGCGGG-3′) primers in a total volume of 25 µL [52]. The first PCR product was amplified using ELPIS (EBT-7861, Seoul, Korea) in a total volume of 20 µL, using primers *Bd*1a (5′-CAGTGTGCCATATGTCACG-3′) and *Bd*2a (5′- CATGGTTCATATCTGTCCAG-3′) to amplify a 300 bp region of ITS1-5.8S-ITS2 [46]. After confirming the product size and quality on a 1% agarose gel, the target band was collected and purified. After TA cloning, it was transformed into *E. coli* DH5α, a plasmid was prepared, and Sanger sequencing was performed. The base sequence obtained was consistent with the base sequence of *Bd* previously studied using the NCBI Basic Local Alignment Search Tool (BLAST version 2.16.0+) [53]. To clarify the evolutionary relationships of the *Bd* lineages detected in this study, their FASTA sequences were retrieved from GenBank via BLAST, and every hit that satisfied the similarity threshold was retained. The sequences were aligned with MUSCLE, and a Maximum-Likelihood tree was constructed in MEGA [54]. Node support was assessed with 1000 bootstrap pseudoreplicates, and the resulting consensus topology was visualized for interpretation. The phylogenetic tree was rooted using two outgroup taxa, *Rhizophlyctis rosea* (accession no. OP799153.1) and *Spizellomyces punctatus* (accession no. PQ552859.1), whose sequences were also retrieved from GenBank.

Similarly, for RV, conventional PCR was performed on 16 of 770 samples that were positive. Referring to the standardized protocol of WHOA, the 625 bp major capsid protein (MCP) 2 was amplified using primers M153 (5′-ATGACCGTCGCCTCATCAC-3′) and M154 (5′-CCATCGAGCCGTTCATGATG-3′) in a total volume of 25 µL [55]. After confirming the size and quality of the target band for the PCR product on a 1% agarose gel, we performed gel purification. After the TA cloning and transformation processes, the DNA base sequence was obtained using Sanger sequencing. Using the NCBI GenBank system, we confirmed that the sequence was consistent with the base sequence of previously studied RV [53]. For phylogenetic analysis, the MCP sequence fasta files of RV from the BLAST GenBank and the base sequences of RV obtained from our sampling were aligned using MEGA software (version 11.0). The phylogenetic tree was generated with MEGA, using the Neighbor-Joining method and 1000 bootstrap replicates to confirm the consistency of the branching pattern [54].

### 2.6. Statistical Analysis

To estimate pathogen prevalence across amphibian species and sampling sites, 95% confidence intervals (CIs) were calculated for the proportion of pathogen-positive individuals relative to the total number of individuals sampled. The 95% CIs were derived using the Wilson score interval method, implemented via the Epitools online calculator; AusVet; https://epitools.ausvet.com.au/ciproportion (accessed on 25 June 2025), with results reported to two decimal places. The corresponding lower and upper 95% CI bounds for all estimates are presented in Supplementary materials.

## 3. Results

### 3.1. Phylogenetic Analysis of Bd and RV Pathogens

**Figure 2:**
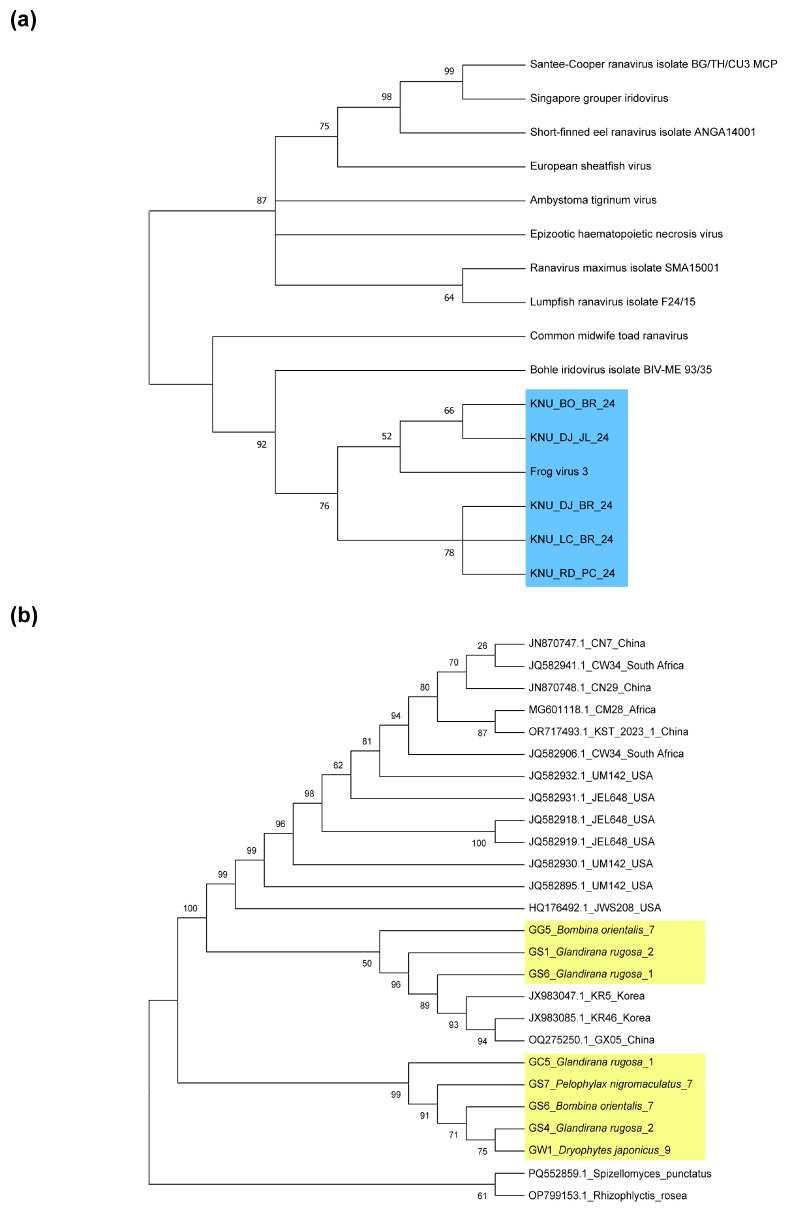
Neighbor joining phylogenetic reconstruction (1000 Ultra-Fast Bootstraps) of (**a**) ranavirus based on MCP, (**b**) *Batrachochytrium dendrobatidis* based on ITS1-5.8S-2 rRNA region. Numbers above or below branches indicate bootstrap values. The *Bd* lineages identified in our study are marked with a yellow background. Also, the RV lineages identified in our study are marked with a blue background.

Based on the Maximum Likelihood (ML) phylogenetic analysis, a total of 24 *Bd* sequences formed a distinct phylogenetic clade, clearly separated from the outgroup taxa (Figure 2). Among the samples collected from our sampling, three lineages (GG5_*Bombina orientalis*_7, GS1_*Glandirana rugosa*_2, and GS6_*Glandirana rugosa*_1) were genetically distinct from one another and formed clades that were closely related to either the Chinese or Korean lineages. In contrast, five lineages (GC5_*Glandirana rugosa*_1, GS7_*Pelophylax nigromaculatus*_7, GS6_*Bombina orientalis*_7, GS4_*Glandirana rugosa*_2, and GW1_*Dryophytes japonicus*_9) were clustered within a separate subgroup, indicating a tight grouping within the same lineage.

In addition, the five analyzed RV isolates (KNU_BO_BR_24, KNU_DJ_JL_24, KNU_DJ_BR_24, KNU_LC_BR_24, KNU_RD_PC_24) were separated from other Iridoviidae lineages obtained from fish and salamanders, and all clustered in an FV3 lineage (Figure 2).

### 3.2. Prevalence of Bd and RV by Species

**Figure 3:**
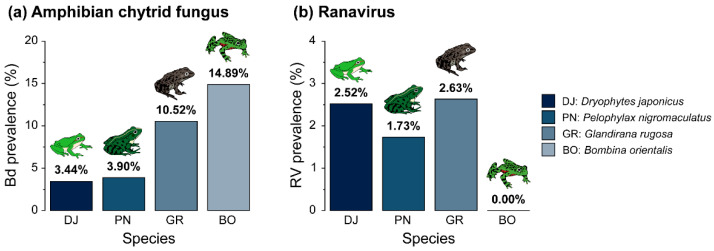
Difference in prevalence in two major pathogens among four anuran species: (**a**) comparison of amphibian chytrid fungus (*Batrachochytrium dendrobatidis*, *Bd*) prevalence among four species; (**b**) comparison of ranavirus (RV) prevalence among four species.

Notably, none of the 752 examined individuals were co-infected with both *Bd* and RV. Furthermore, detailed raw data organized by region and species are available in Supplementary materials. The prevalence of *Bd* varied significantly among species. The overall mean *Bd* prevalence across the four species was 4.65% (0.03, 0.06). *D. japonicus* 3.44% (0.02, 0.06) and *P. nigromaculatus* 3.90% (0.02, 0.07) exhibited a lower *Bd* prevalence than *G. rugosa* 10.52% (0.04–0.24) and *B. orientalis* 14.89% (0.07, 0.28) (Figure 3a).

The overall prevalence of RV across the four species was 2.13% (0.01, 0.03), which was lower than that of *Bd*. Unlike *Bd*, *D. japonicus* 2.52% (0.01, 0.04) and *G. rugosa* 2.63% (0.00, 0.13) exhibited higher RV prevalence than *P. nigromaculatus* 1.73% (0.01, 0.04), and no RV-positive individuals were detected in *B. orientalis* (Figure 3b).

### 3.3. Distribution of Bd Throughout the Study Area

**Figure 4:**
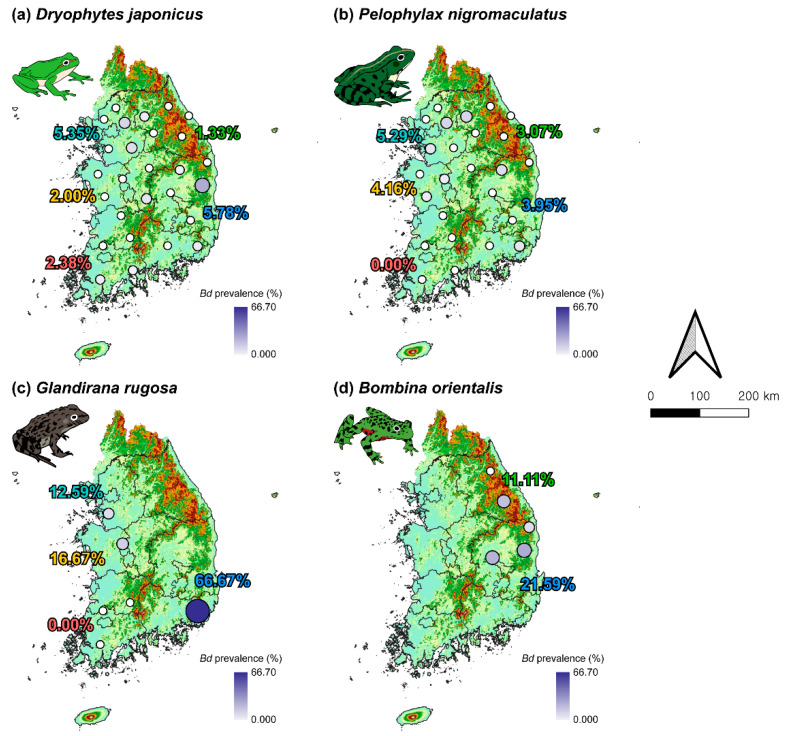
National distribution of *Bd* prevalence among four anuran species in South Korea. Regions are color-coded as follows: Gyeonggi-do (light blue), Gangwon-do (green), Chungcheong-do (yellow), Jeolla-do (red), and Gyeongsang-do (blue). The percentage labels by region represent the overall *Bd* prevalence for each species within that region. Darker gradient colors and larger circle sizes both indicate higher prevalence levels.

In *D. japonicus*, the prevalence of *Bd* was detected in 9 of 27 sites (Figure 4a). The highest prevalence of *Bd* in *D. japonicus* was observed in Gyeongsang-do (5.78%), with the GS4 site (28.5%) exhibiting the highest prevalence. This was followed by Gyeonggi-do, where the overall prevalence reached 5.35%, and high prevalence was recorded at GG3 (14.29%) and GG1 (12.50%). Notably, these two sites are adjacent to GW4 (6.67%) in Gangwon-do, the only site in that region where *Bd* was detected. Interestingly, the prevalence of *Bd* was relatively evenly distributed across southern coastal sites, including JL1 (5.26%) and JL2 (6.67%) in Jeolla-do, and GS1 (6.67%) and GS4 in Gyeongsang-do.

In *P. nigromaculatus*, the prevalence of *Bd* was observed at 7 of the 25 sites where the species was collected (Figure 4b). Gyeonggi-do showed the highest overall prevalence (5.29%), with GG3 (15.38%) and GW4 (15.38%) exhibiting the highest site-specific prevalence, suggesting a similar spatial pattern of *Bd* hotspots as seen in *D. japonicus*. GS1 (11.1%) in Gyeongsang-do, which showed a relatively high prevalence, was also one of the sites with the highest prevalence of *Bd* in *D. japonicus*. Likewise, GS6 (12.5%) matched a site where the prevalence of *Bd* was also detected in *D. japonicus*. In contrast, *Bd* was not detected in any *P. nigromaculatus* individuals sampled from the three sites in Jeolla-do.

*G. rugosa* was sampled from all sites except Gangwon-do, and the prevalence of *Bd* was detected at GG2 (12.5%) in Gyeonggi-do, CC3 (16.66%) in Chungcheong-do, and GS1 (66.67%) in Gyeongsang-do (Figure 4c). In particular, GS1 in Gyeongsang-do, where both *D. japonicus* and *P. nigromaculatus* were present, showed the highest prevalence of *Bd* of all the other sites. In addition, GG2 and CC3 showed a relatively high prevalence of *Bd*, similar to the sites where the prevalence of *Bd* in *P. nigromaculatus* was detected. Similar to *P. nigromaculatus*, not a single individual from the three sites in Jeolla-do was found to be *Bd*-positive.

*B. orientalis* was collected only from Gangwon-do and Gyeongsang-do and showed an overall high prevalence of *Bd* (Figure 4d). In Gangwon-do, no *Bd* was detected at GW5 (0%), but GW1 (22.22%) had a high prevalence of *Bd*. In Gyeongsang-do, GS4 (27.27%) exhibited the highest prevalence of *Bd*, which corresponds to the site with the highest prevalence of *Bd* in *D. japonicus* as well, indicating this site as a potential hotspot for infection. On the other hand, sites such as GW1 in Gangwon-do and GS5 (25%) and GS6 (12.5%) in Gyeongsang-do were showed high prevalence of *Bd* in *B. orientalis* but no evidence of infection in *D. japonicus* or *P. nigromaculatus*.

### 3.4. Distribution of RVs Throughout the Study Area

**Figure 5:**
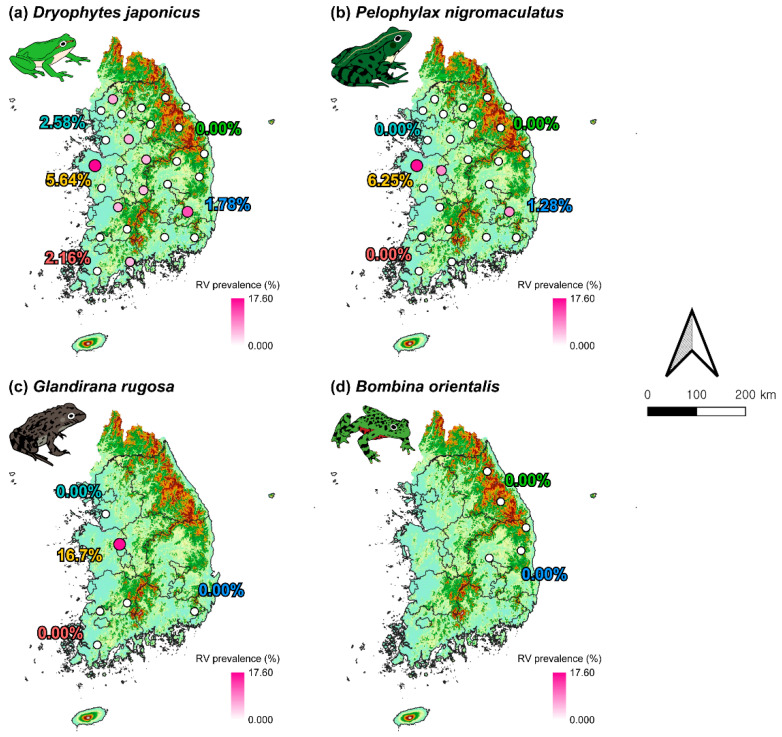
National distribution of RV prevalence among four anuran species in South Korea. Regions are color-coded as follows: Gyeonggi-do (light blue), Gangwon-do (green), Chungcheong-do (yellow), Jeolla-do (red), and Gyeongsang-do (blue). The percentage labels by region represent the overall RV prevalence for each species within that region. Darker gradient colors and larger circle sizes both indicate higher prevalence levels.

*D. japonicus* showed a prevalence of RV at only 7 out of 27 sites (Figure 5a). The highest prevalence of RV was detected in Chungcheong-do (5.64%), with site CC4 (17.65%) showing the highest prevalence of RV across all sites. Although Gyeongsang-do (1.78%) exhibited a relatively low overall prevalence of RV, site GS3 (12.5%) displayed the second-highest prevalence of RV among all surveyed locations.

*P. nigromaculatus* exhibited the prevalence of RV at only 3 of the 25 sites, with no prevalence of RV found in Gyeonggi-do, Gangwon-do, or Jeolla-do (Figure 5b). Similar to *D. japonicus*, the highest prevalence of RV was found in Chungcheong-do (6.25%), particularly at site CC4 (16.67%), followed by site CC3 (8.33%). Likewise, although the overall prevalence of RV in Gyeongsang-do (1.28%) was low, site GS3 (7.69%) showed a relatively high prevalence of RV, consistent with the pattern observed in *D. japonicus* at the same location.

For *G. rugosa*, the prevalence of RV was detected only at site CC3 (16.67%) in Chungcheong-do, which also showed the second-highest prevalence of RV in *P. nigromaculatus*. In contrast, no prevalence of RV was observed in populations at any other sites. Prevalence of RV was not detected in any individuals of *B. orientalis* (Figure 5d).

### 3.5. Pathogen Prevalences by Difference in Sex and Life History

**Figure 6:**
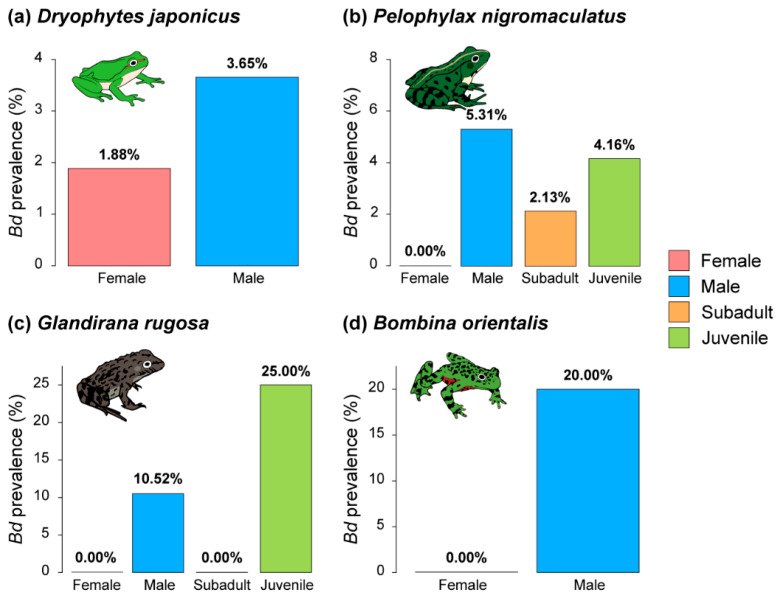
Comparison of *Bd* prevalence by sex and life histories from four anuran species: (**a**) the prevalence of *Bd* in Japanese tree frogs (*Dryophytes japonicus*), (**b**) the prevalence of *Bd* in black-spotted pond frogs (*Pelophylax nigromaculatus*), (**c**) the prevalence of *Bd* in Japanese wrinkled frogs (*Glandirana rugosa*), and (**d**) the prevalence of *Bd* in oriental fire-bellied toads (*Bombina orientalis*). *D. japonicus* and *B. orientalis* had no younger life stages represented in our captures.

In all four species, *Bd* was more prevalent in males than females (Figure 6). In particular, in *P. nigromaculatus*, the prevalence was highest in male frogs, followed by juvenile and subadult frogs. In contrast, in *G. rugosa*, the prevalence was highest in juvenile frogs, followed by male frogs, and there was no prevalence in female or subadult frogs.

**Figure 7:**
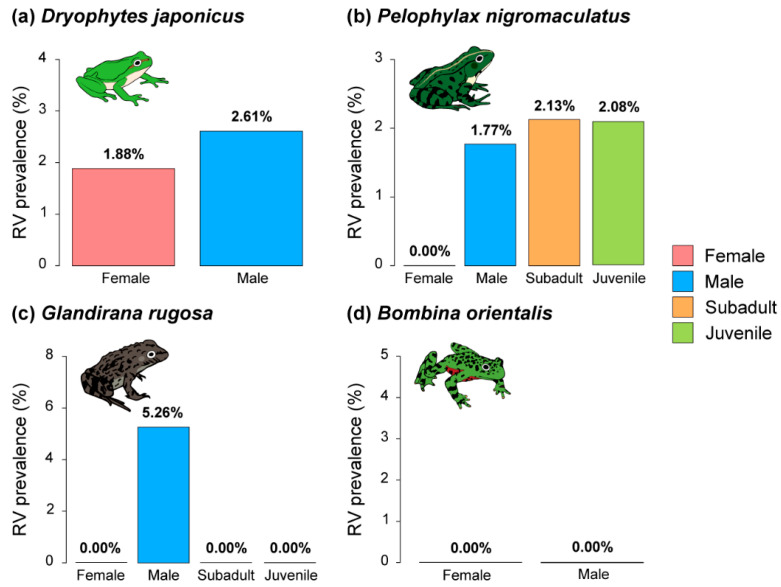
Comparison of RV prevalence by sex and life histories from four anuran species: (**a**) the prevalence of RV in Japanese tree frogs (*Dryophytes japonicus*), (**b**) the prevalence of RV in black-spotted pond frogs (*Pelophylax nigromaculatus*), (**c**) the prevalence of RV in Japanese wrinkled frogs (*Glandirana rugosa*), and (**d**) the prevalence of RV in oriental fire-bellied toads (*Bombina orientalis*). *D. japonicus* and *B. orientalis* had no younger life stages represented in our captures.

Except for *B. orientalis*, which had no individuals with an RV infection, RV was more prevalent in males than in females for the other species (Figure 7). However, in contrast to the results for *Bd*, juvenile and subadult *P. nigromaculatus* frogs showed higher RV prevalence than male frogs. In contrast, in *G. rugosa*, only male frogs showed RV prevalence.

### 3.6. Co-Occurrence of Bd and RV in the Study Area

**Figure 8:**
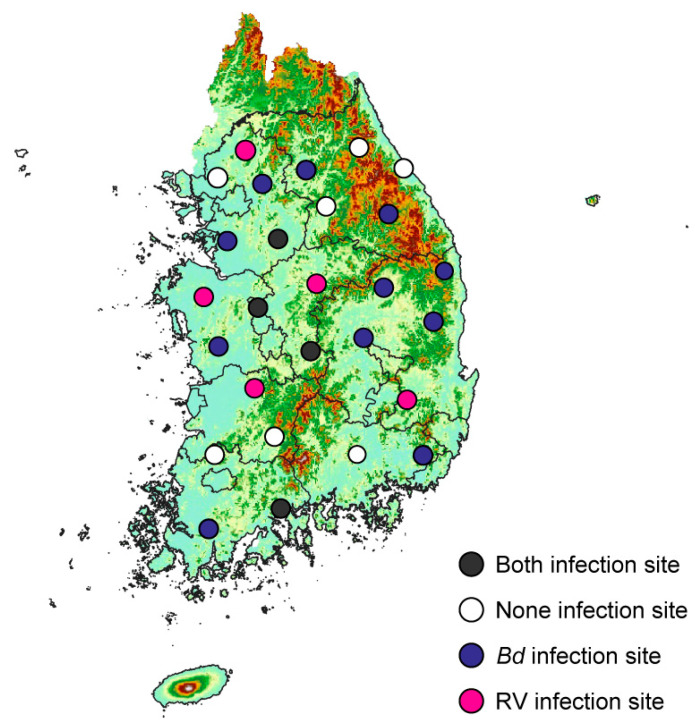
Summary of the South Korean national distribution of *Bd* and RV infection from four anuran species. Black circles indicate sites with simultaneous infections of both *Bd* and RV, and white circles indicate sites with detection of neither disease. Purple circles represent sites with only *Bd* infection, and pink circles represent sites with only RV infection.

Across all species, *Bd* was detected at 15 of the 27 sites surveyed, whereas RV was present at only 9 sites. Both *Bd* and RV were simultaneously detected at 4 sites, and neither pathogen was detected at 7 sites (Figure 8).

Of the four sites (GG1, CC1, CC3, JL1) where both *Bd* and RV were simultaneously detected, three were located in Gyeonggi-do and Chungcheong-do, regions where both pathogens were more commonly observed. In contrast, Gangwon-do and Jeolla-do have relatively many sites with no detection of either pathogen. Specifically, *Bd* and RV were not detected at three sites in Gangwon-do (GW2, GW3, and GW5) and two sites in Jeolla-do (JL3 and JL4).

In Gyeonggi-do, *Bd* was detected without RV at GG2 and GG3, while RV alone was found at GG5. In Chungcheong-do, all sampling sites showed at least one pathogen. *Bd* was detected alone at CC2, while RV was detected alone at CC4 and CC5. Gangwon-do was relatively free of both pathogens. None of the sites showed RV detection, and therefore, no coinfection occurred. *Bd* alone was detected at GW1 and GW4. In Jeolla-do, *Bd* was detected alone at JL2, and RV was detected alone at JL5. In Gyeongsang-do, no sites showed simultaneous detection of both pathogens. *Bd* alone was much more widespread than RV, being found at five sites (GS1, GS4, GS5, GS6, and GS7), whereas RV alone was detected at only one site (GS3).

## 4. Discussion

In this study, we analyzed differences in the prevalence of two major pathogens, *Bd* and RV, in anurans according to species, region, sex, and life history. We also identified areas where the co-occurrence of these two pathogens is densely concentrated.

*Bd* and RV have aquatic life cycles and mostly spread to host amphibians via water or other moist environments [18,19]. Therefore, it can be expected that terrestrial species may show lower prevalence than semi-aquatic species that are not predominantly independent of water. For example, among the four species, *G. rugosa* is a semi-aquatic frog and wintering anuran [56,57] that does not leave water for much of its life cycle. In addition, because they often hibernate in a tadpole state, they prefer permanent wetlands that do not dry out during the winter, and such places have been shown to have higher *Bd* infection rates than temporal wetlands [19]. However, our results showed that the species-specific prevalence differences were not significantly related to the patterns of microhabitat use of the species. Although the semi-aquatic species *G. rugosa* and *B. orientalis* showed high *Bd* prevalence, the semi-aquatic species *P. nigromaculatus* showed a similar prevalence to the terrestrial species *D. japonicus*. The species-specific prevalence differences for RV also showed that *D. japonicus* and *G. rugosa* showed similar prevalence, whereas *P. nigromaculatus* showed lower prevalence, and *B. orientalis* showed no prevalence at all. Although *G. rugosa* and *B. orientalis* were not evenly collected across all sites, these results were not significantly different between *P. nigromaculatus* and *D. japonicus*. These prevalences varied more between the collection sites than between species. Similarly, the prevalence of *Bd* or RV can be significantly influenced by the geographical characteristics, season, and surrounding environment of the species distribution [19,25], but the ecological differences of the species cannot be ignored. Because the spread and epidemiology of the pathogen can be strongly influenced by the ecological, behavioral, and physiological characteristics of the species, it is necessary to understand the variation in prevalence according to the ecological characteristics of specific species in detail rather than at a nationwide scale.

The representative differences in prevalence according to sex and life history support this hypothesis. Both *Bd* and RV showed a higher prevalence in male frogs than in female frogs. Previous studies have reported higher rates of pathogen and parasitic infections in male frogs [31,58,59]. Male frogs spend more time fulfilling their reproductive strategies and take greater risks than females, such as territory defense, vocalization, and protection [33,60]. These reproductive behaviors can attract predators and pests such as bats and midges [61]. Recent studies have reported that insects such as midges and mosquitoes may have the capacity to transmit *Bd* to uninfected hosts, and that high exposure to these vectors may play a secondary role in increasing pathogen prevalence [62,63]. In contrast, in most species, females may have lower prevalence rates because they experience relatively lower exposure risk by selecting mates and remaining at breeding sites for shorter periods than males, who continue calling even after oviposition [64].

However, given that life-history circumstances differ according to sex, the results were inconsistent. In *P. nigromaculatus*, the prevalence of *Bd* was higher in males than in juveniles, whereas in *G. rugosa*, the prevalence was higher in juveniles than in adult males. In contrast, RV showed the opposite result to *Bd*. In *P. nigromaculatus*, the prevalence of RV was higher in juvenile frogs, whereas in *G. rugosa*, only adult males showed a higher RV prevalence. *Bd* may be more threatening to juveniles than to adult frogs. In response to thyroid hormones, *Bd* can infect frogs that metamorphose during this period and are already exposed to infections before they emerge on land [30]. RV has been reported to be the most severe in tadpoles, with a higher prevalence in juveniles than in adults [65]. Our results suggest that this general pattern may vary according to species and season. *P. nigromaculatus* was sampled at or just after the peak of its breeding season, whereas *G. rugosa* was sampled at or just after the beginning of its breeding season. Furthermore, *P. nigromaculatus* is a terrestrial hibernator, whereas *G. rugosa* is an aquatic hibernator that hibernates in gently flowing water; individuals that hibernate as tadpoles may emerge in spring to metamorphose. Thus, we expect that the combined effects of species-specific differences in the peak reproductive season, hibernation strategy, timing of metamorphosis, and life cycle of the pathogen may have led to differences in the prevalence of the two pathogens across life histories. However, age and sex-based comparisons were not conducted across all species in this study. To enhance comparative analyses of prevalence by age and sex, future research should incorporate more balanced sampling of age classes for each species.

In South Korea, *Bd* and RV tend to be concentrated in the central region of the nation. Most *Bd* was concentrated in the mountainous and coastal areas of Gangwon and Gyeongsang-do, whereas RV was concentrated in the relatively flat central region. *Bd* is generally intolerant of high temperatures and tends to be activated at low temperatures [34]. Therefore, *Bd* is widely distributed in mountainous and coastal areas [66]. Because of these characteristics, *Bd* often has a more severe impact on amphibians living at higher altitudes or in cold environments [67]. In contrast, RV is more active at higher temperatures [68]. There is evidence that these characteristics may increase the prevalence of RV as the temperature increases [14]. Although our results suggest that the distributions of *Bd* and RV reflect these characteristics, this is not certain. Similar to previous nationwide investigations [11,69], we believe that these two pathogens were evenly distributed throughout the country. The results of our study may have been due to seasonal changes in distribution. During summer, when temperatures are high, the prevalence of RV may be higher nationwide. Whereas during spring and fall temperatures are lower, the prevalence of *Bd* may increase. Therefore, locations where *Bd* and RV are concentrated are likely to have seasonal distribution patterns rather than pathogen hotspots.

Interestingly, *Bd* prevalence in neighboring countries has been reported at varying levels. In China, which shares similar seasonal and climatic patterns with the Korean Peninsula, approximately 7.5% (157/2075) of sampled individuals tested positive for *Bd* [70], and RV infection was confirmed in 8.5% (92/1076) of individuals [71]. In Japan, *Bd* was detected in 4.1% (87/2103) of wild amphibians [52], showing a prevalence rate comparable to that of our study. While China exhibited a slightly higher prevalence of both pathogens, Japan showed a similar infection rate to Korea, despite comparable ecological and climatic conditions across these regions. In addition, a coinfection of *Bd* and RV was reported in 0.39% (2/501) of sampled individuals in the Guangxi Zhuang Autonomous Region (GAR) in China, indicating the possibility of multi-pathogen interactions in natural populations. In contrast, our study did not detect any coinfected individuals, nor did we observe any spatial clustering of coinfection, despite both pathogens being present at the same sites. This discrepancy may be attributable to the limited temporal scope of our sampling. The coinfection data from China may reflect cumulative prevalence across multiple seasons, which our study could not capture. Therefore, it is necessary to predict the movement of *Bd* and RV prevalence by monitoring them in all four seasons or at a surveillance across broader spatial and seasonal scales. These results, when combined with studies on the species-specific characteristics of many species, can help understand pathogen epidemiology and systematic disease management.

## 5. Conclusions

Our study provides the first comprehensive investigation of the simultaneous occurrence of *Batrachochytrium dendrobatidis* and ranavirus in amphibian populations across South Korea. The results demonstrate that pathogen prevalence is more strongly influenced by geographic region than by host species, indicating the dominant role of environmental variables and seasonal dynamics in shaping pathogen distribution. While semi-aquatic species occasionally exhibited higher infection rates, the overall relationship between microhabitat use and pathogen prevalence was inconsistent, suggesting the involvement of complex ecological and physiological factors. Notable differences in prevalence by sex and life history stage further emphasize the significance of behaviorally mediated exposure and species-specific phenology in driving infection patterns. In particular, investigating the interactions between *Bd* and RV under various temperature conditions through simultaneous exposure experiments could provide valuable insights into the mechanisms governing pathogen coexistence or exclusion. These findings highlight the necessity of long-term, seasonally resolved surveillance to improve understanding of disease ecology and to guide evidence-based amphibian conservation and management strategies.

## Figures and Tables

**Table 1:** The sampling date and number of individuals per species at each collection site: five sites in Gyeonggi-do (GG1–GG5), five sites in Gangwon-do (GW1–GW5), five sites in Chungcheong-do (CC1–CC5), five sites in Jeolla-do (JL1–JL5), and seven sites in Gyeongsang-do (GS1–GS5).

Sites	Sampling Date	Sampling Temperature/Humidity	Number of Samples			
			Bombinatoridae	Hylidae	Ranidae	Ranidae
			*Bombina orientalis*	*Dryophytes japonicus*	*Glandirana rugosa*	*Pelophylax nigromaculatus*
GG1	May 28	18.3 °C/56%	-	16	-	10
GG2	May 28	18.6 °C/62%	-	21	8	9
GG3	May 23	20.9 °C/63%	-	14	-	13
GG4	May 23	19.2 °C/78%	-	15	-	10
GG5	May 23	18.8 °C/66%	-	15	-	15
GW1	May 28	11.8 °C/77%	9	14	-	9
GW2	June 03	20.6 °C/45%	-	21	-	7
GW3	June 03	15.3 °C/88%	-	18	-	3
GW4	June 03	20.6 °C/32%	-	15	-	13
GW5	June 03	17.3 °C/57%	15	15	-	9
CC1	June 06	22.4 °C/57%	-	20	-	-
CC2	June 07	21.8 °C/57%	-	14	-	12
CC3	June 07	22.5 °C/66%	-	12	6	12
CC4	June 06	16.9 °C/61%	-	17	-	6
CC5	June 06	19.1 °C/59%	-	18	-	7
JL1	May 31	22.6 °C/73%	-	19	-	7
JL2	May 31	19.5 °C/71%	-	15	8	6
JL3	May 31	20.4 °C/53%	-	15	6	7
JL4	May 31	18.6 °C/70%	-	13	7	11
JL5	May 31	18.2 °C/69%	-	18	-	11
GS1	May 31	21.5 °C/67%	-	15	3	9
GS2	May 28	18.8 °C/64%	-	20	-	2
GS3	May 28	17.6 °C/69%	-	16	-	13
GS4	May 28	17.1 °C/65%	11	14	-	-
GS5	May 23	19.6 °C/54%	4	15	-	9
GS6	May 23	23.4 °C/45%	8	12	-	8
GS7	May 23	22.3 °C/52%	-	19	-	8

## Data Availability

The data presented in this study are available on request from the corresponding author.

## Supplementary Materials

The following supporting information can be downloaded at: https://www.mdpi.com/article/10.3390/ani15142132/s1.

## References

[1] Luedtke J.A., Chanson J., Neam K., Hobin L., Maciel A.O., Catenazzi A., Borzée A., Hamidy A., Aowphol A., Jean A. (2023). Ongoing declines for the world’s amphibians in the face of emerging threats. Nature.

[2] Gray M.J., Miller D.L., Hoverman J.T. (2009). Ecology and pathology of amphibian ranaviruses. Dis. Aquat. Organ..

[3] Murray K.A., Skerratt L.F., Speare R., McCALLUM H. (2009). Impact and dynamics of disease in species threatened by the amphibian chytrid fungus, *Batrachochytrium dendrobatidis*. Conserv. Biol..

[4] Weinstein S.B. (2009). An aquatic disease on a terrestrial salamander: Individual and population level effects of the amphibian chytrid fungus, *Batrachochytrium dendrobatidis*, on *Batrachoseps attenuatus* (Plethodontidae). Copeia.

[5] Price S.J., Garner T.W., Nichols R.A., Balloux F., Ayres C., de Alba A.M.-C., Bosch J. (2014). Collapse of amphibian communities due to an introduced Ranavirus. Curr. Biol..

[6] Fisher M.C., Garner T.W. (2020). Chytrid fungi and global amphibian declines. Nat. Rev. Microbiol..

[7] Campbell C.R., Voyles J., Cook D.I., Dinudom A. (2012). Frog skin epithelium: Electrolyte transport and chytridiomycosis. Int. J. Biochem. Cell Biol..

[8] Docherty D.E., Meteyer C.U., Wang J., Mao J., Case S.T., Chinchar V.G. (2003). Diagnostic and molecular evaluation of three iridovirus–associated salamander mortality events. J. Wildl. Dis..

[9] Scheele B.C., Pasmans F., Skerratt L.F., Berger L., Martel A., Beukema W., Acevedo A.A., Burrowes P.A., Carvalho T., Catenazzi A. (2019). Amphibian fungal panzootic causes catastrophic and ongoing loss of biodiversity. Science.

[10] Bosch J., Monsalve-Carcaño C., Price S.J., Bielby J. (2020). Single infection with *Batrachochytrium dendrobatidis* or Ranavirus does not increase probability of co–infection in a montane community of amphibians. Sci. Rep..

[11] Bataille A., Fong J.J., Cha M., Wogan G.O., Baek H.J., Lee H., Min M.S., Waldman B. (2013). Genetic evidence for a high diversity and wide distribution of endemic strains of the pathogenic chytrid fungus *Batrachochytrium dendrobatidis* in wild Asian amphibians. Mol. Ecol..

[12] Roh N., Park J., Kim J., Kwon H., Park D. (2022). Prevalence of Ranavirus infection in three anuran species across South Korea. Viruses.

[13] Berger L., Speare R., Hines H., Marantelli G., Hyatt A., McDonald K., Skerratt L., Olsen V., Clarke J., Gillespie G. (2004). Effect of season and temperature on mortality in amphibians due to chytridiomycosis. Aust. Vet. J..

[14] Brand M.D., Hill R.D., Brenes R., Chaney J.C., Wilkes R.P., Grayfer L., Miller D.L., Gray M.J. (2016). Water temperature affects susceptibility to ranavirus. EcoHealth.

[15] Knapp R.A., Briggs C.J., Smith T.C., Maurer J.R. (2011). Nowhere to hide: Impact of a temperature-sensitive amphibian pathogen along an elevation gradient in the temperate zone. Ecosphere.

[16] Cortazar-Chinarro M., Richter-Boix A., Rödin-Mörch P., Halvarsson P., Logue J., Laurila A., Höglund J. (2024). Association between the skin microbiome and MHC class II diversity in an amphibian. Mol. Ecol..

[17] Lau Q., Igawa T., Kosch T.A., Dharmayanthi A.B., Berger L., Skerratt L.F., Satta Y. (2023). Conserved evolution of MHC supertypes among Japanese frogs suggests selection for *Bd* resistance. Animals.

[18] Robert J., George E., Andino F.D.J., Chen G. (2011). Waterborne infectivity of the Ranavirus frog virus 3 in *Xenopus laevis*. Virology.

[19] Ruggeri J., de Carvalho-e-Silva S.P., James T.Y., Toledo L.F. (2018). Amphibian chytrid infection is influenced by rainfall seasonality and water availability. Dis. Aquat. Org..

[20] Brunner J.L., Storfer A., Gray M.J., Hoverman J.T. (2015). Ranavirus ecology and evolution: From epidemiology to extinction. Ranaviruses: Lethal Pathogens of Ectothermic Vertebrates.

[21] Robinson C.W., McNulty S.A., Titus V.R. (2018). No safe space: Prevalence and distribution of Batrachochytrium dendrobatidis in amphibians in a highly-protected landscape. Herpetol. Conserv. Biol..

[22] Gould J., Valdez J., Stockwell M., Clulow S., Mahony M. (2019). Mosquitoes as a potential vector for the transmission of the amphibian chytrid fungus. Preprints.

[23] Brunner J.L., Yarber C.M. (2018). Evaluating the importance of environmental persistence for Ranavirus transmission and epidemiology. Adv. Virus Res..

[24] Park J.-K., Do Y. (2024). Developmental temperature modulates microplastics impact on amphibian life history without affecting ontogenetic microplastic transfer. J. Hazard. Mater..

[25] Hoverman J.T., Gray M.J., Haislip N.A., Miller D.L. (2011). Phylogeny, life history, and ecology contribute to differences in amphibian susceptibility to ranaviruses. EcoHealth.

[26] Haislip N.A., Gray M.J., Hoverman J.T., Miller D.L. (2011). Development and disease: How susceptibility to an emerging pathogen changes through anuran development. PLoS ONE.

[27] Gray M.J., Gregory Chinchar V. (2015). Introduction: History and Future of Ranaviruses. Ranaviruses.

[28] Warne R.W., Crespi E.J., Brunner J.L. (2011). Escape from the pond: Stress and developmental responses to ranavirus infection in wood frog tadpoles. Funct. Ecol..

[29] Green D.E., CoNVerSe K.A., SCHrADer A.K. (2002). Epizootiology of sixty-four amphibian morbidity and mortality events in the USA, 1996–2001. Ann. N. Y. Acad. Sci..

[30] Thekkiniath J.C., Zabet-Moghaddam M., San Francisco S.K., San Francisco M.J. (2013). A novel subtilisin–like serine protease of *Batrachochytrium dendrobatidis* is induced by thyroid hormone and degrades antimicrobial peptides. Fungal Biol..

[31] Adams A.J., Kupferberg S.J., Wilber M.Q., Pessier A.P., Grefsrud M., Bobzien S., Vredenburg V.T., Briggs C.J. (2017). Extreme drought, host density, sex, and bullfrogs influence fungal pathogen infection in a declining lotic amphibian. Ecosphere.

[32] Ryser J. (1989). Weight loss, reproductive output, and the cost of reproduction in the common frog, *Rana temporaria*. Oecologia.

[33] Bevier C.R. (1997). Utilization of energy substrates during calling activity in tropical frogs. Behav. Ecol. Sociobiol..

[34] Piotrowski J.S., Annis S.L., Longcore J.E. (2004). Physiology of *Batrachochytrium dendrobatidis*, a chytrid pathogen of amphibians. Mycologia.

[35] La Fauce K., Ariel E., Munns S., Rush C., Owens L. (2012). Influence of temperature and exposure time on the infectivity of Bohle iridovirus, a ranavirus. Aquaculture.

[36] AmphibiaWeb Hyla japonica: Japanese Tree Frog. https://amphibiaweb.org/species/832.

[37] AmphibiawWeb Bombina orientalis: Oriental Fire-Bellied Toad. https://amphibiaweb.org/species/2045.

[38] AmphibiaWeb Pelophylax nigromaculatus: Dark-Spotted Frog. https://amphibiaweb.org/species/5109.

[39] AmphibiaWeb Glandirana rugosa: Wrinkled Frog. https://amphibiaweb.org/species/5138.

[40] Boyle A.H.D., Olsen V., Boyle D., Berger L., Obendorf D., Dalton A., Kriger K., Hero M., Hines H., Phillott R. (2007). Diagnostic assays and sampling protocols for the detection of *Batrachochytrium dendrobatidis*. Dis. Aquat. Org..

[41] Archard G.A., Goldsmith A. (2010). Euthanasia methods, corticosterone and haematocrit levels in *Xenopus laevis*: Evidence for differences in stress?. Anim. Welf..

[42] Balko J.A., Posner L.P., Chinnadurai S.K. (2019). Immersion in tricaine methanesulfonate (MS–222) is not sufficient for euthanasia of smokey jungle frogs (*Leptodactylus pentadactylus*). J. Zoo Wildl. Med..

[43] Navarro K., Jampachaisri K., Chu D., Pacharinsak C. (2022). Bupivacaine as a euthanasia agent for African Clawed Frogs (*Xenopus laevis*). PLoS ONE.

[44] Park J., Grajal-Puche A., Roh N.-H., Park I.-K., Ra N.-Y., Park D. (2021). First detection of ranavirus in a wild population of Dybowski’s brown frog (*Rana dybowskii*) in South Korea. J. Ecol. Environ..

[45] Boyle D.G., Boyle D., Olsen V., Morgan J., Hyatt A. (2004). Rapid quantitative detection of chytridiomycosis (*Batrachochytrium dendrobatidis*) in amphibian samples using real–time Taqman PCR assay. Dis. Aquat. Org..

[46] Gaertner J.P., Forstner M.R., O’Donnell L., Hahn D. (2009). Detection of *Batrachochytrium dendrobatidis* in endemic salamander species from Central Texas. EcoHealth.

[47] Annis S.L., Dastoor F.P., Ziel H., Daszak P., Longcore J.E. (2004). A DNA–based assay identifies *Batrachochytrium dendrobatidis* in amphibians. J. Wildl. Dis..

[48] WOAH—World Organisation for Animal Health (2019). Manual of Diagnostic Tests for Aquatic Animals—Chapter 2. 1. 1. Infection with *Batrachochytrium dendrobatidis*. https://www.woah.org/en/disease/chytridiomycosis-batrachochytrium-dendrobatidis/.

[49] Stilwell N.K., Whittington R.J., Hick P.M., Becker J.A., Ariel E., Van Beurden S., Vendramin N., Olesen N.J., Waltzek T.B. (2018). Partial validation of a TaqMan real–time quantitative PCR for the detection of ranaviruses. Dis. Aquat. Org..

[50] Tan W.G., Barkman T.J., Chinchar V.G., Essani K. (2004). Comparative genomic analyses of frog virus 3, type species of the genus Ranavirus (family Iridoviridae). Virology.

[51] Karwacki E.E., Atkinson M.S., Ossiboff R.J., Savage A.E. (2018). Novel quantitative PCR assay specific for the emerging Perkinsea amphibian pathogen reveals seasonal infection dynamics. Dis. Aquat. Org..

[52] Goka K., Yokoyama J., Une Y., Kuroki T., Suzuki K., Nakahara M., Kobayashi A., Inaba S., Mizutani T., Hyatt A.D. (2009). Amphibian chytridiomycosis in Japan: Distribution, haplotypes and possible route of entry into Japan. Mol. Ecol..

[53] Camacho C., Coulouris G., Avagyan V., Ma N., Papadopoulos J., Bealer K., Madden T.L. (2009). BLAST+: Architecture and applications. BMC Bioinform..

[54] Tamura K., Stecher G., Kumar S. (2021). MEGA11: Molecular evolutionary genetics analysis version 11. Mol. Biol. Evol..

[55] WOAH—World Organisation for Animal Health (2021). Manual of Diagnostic Tests for Aquatic Animals—Chapter 2. 1. 3. Infection with Ranavirus. https://www.woah.org/en/document/ranaviruses-infection-with/.

[56] Lee J.-E., Park J.-K., Do Y. (2024). Gut microbiome diversity and function during hibernation and spring emergence in an aquatic frog. PLoS ONE.

[57] PARK J.K., Do Y. (2024). The difference and variation of gut bacterial community and host physiology can support adaptation during and after overwintering in frog population. Integr. Zool..

[58] Klein S.L. (2004). Hormonal and immunological mechanisms mediating sex differences in parasite infection. Parasite Immunol..

[59] Kelehear C., Brown G.P., Shine R. (2012). Size and sex matter: Infection dynamics of an invading parasite (the pentastome *Raillietiella frenatus*) in an invading host (the cane toad *Rhinella marina*). Parasitology.

[60] Titon S.C.M., de Assis V.R., Junior B.T., Barsotti A.M.G., Flanagan S.P., Gomes F.R. (2016). Calling rate, corticosterone plasma levels and immunocompetence of Hypsiboas albopunctatus. Comp. Biochem. Physiol. Part A Mol. Integr. Physiol..

[61] Halfwerk W., Blaas M., Kramer L., Hijner N., Trillo P.A., Bernal X.E., Page R.A., Goutte S., Ryan M.J., Ellers J. (2019). Adaptive changes in sexual signalling in response to urbanization. Nat. Ecol. Evol..

[62] Toledo L.F., Ruggeri J., Leite Ferraz de Campos L., Martins M., Neckel-Oliveira S., Breviglieri C.P.B. (2021). Midges not only sucks, but may carry lethal pathogens to wild amphibians. Biotropica.

[63] Reinhold J.M., Halbert E., Roark M., Smith S.N., Stroh K.M., Siler C.D., McLeod D.S., Lahondère C. (2023). The role of Culex territans mosquitoes in the transmission of *Batrachochytrium dendrobatidis* to amphibian hosts. Parasites Vectors.

[64] Gittins S. (1983). The breeding migration of the Common toad (*Bufo bufo*) to a pond in mid-Wales. J. Zool..

[65] Cullen B., Owens L. (2002). Experimental challenge and clinical cases of Bohle iridovirus (BIV) in native Australian anurans. Dis. Aquat. Org..

[66] Fellers G.M., Cole R.A., Reinitz D.M., Kleeman P.M. (2011). Amphibian chytrid fungus (*Batrachochytrium dendrobatidis*) in coastal and montane California, USA anurans. Herpetol. Conserv. Biol..

[67] Gründler M.C., Toledo L.F., Parra-Olea G., Haddad C.F., Giasson L.O., Sawaya R.J., Prado C.P., Araujo O.G., Zara F.J., Centeno F.C. (2012). Interaction between breeding habitat and elevation affects prevalence but not infection intensity of *Batrachochytrium dendrobatidis* in Brazilian anuran assemblages. Dis. Aquat. Org..

[68] Ariel E., Nicolajsen N., Christophersen M.-B., Holopainen R., Tapiovaara H., Jensen B.B. (2009). Propagation and isolation of ranaviruses in cell culture. Aquaculture.

[69] Roh N.-H., Kim J., Park J., Park D. (2023). High ranavirus infection rates at low and extreme temperatures in the tadpoles of Japanese treefrogs (*Dryophytes japonicus*) that breed in rice paddies in the summer. J. Ecol. Environ..

[70] Zhou L., Gratwicke B., Wang J., Guo Z., Zhang M., Wang C., Hou M., Xu T., Wu H., Jin T. (2023). Global and endemic Asian lineages of the emerging pathogenic fungus *Batrachochytrium dendrobatidis* widely infect amphibians in China. Sci. Rep..

[71] Herath J., Sun D., Ellepola G., Subramaniam K., Meegaskumbura M. (2023). Emerging threat of ranavirus: Prevalence, genetic diversity, and climatic drivers of *Ranavirus* (*Iridoviridae*) in ectothermic vertebrates of Asia. Front. Vet. Sci..

