# Correction: Lee et al. Identification of Simultaneous Occurrence of Amphibian Chytrid Fungi and Ranavirus in South Korea. *Animals* 2025, *15*, 2132

**DOI:** 10.3390/ani16020309

**Published:** 2026-01-20

**Authors:** Ji-Eun Lee, Young Jin Park, Mun-Gyeong Kwon, Yun-Kyeong Oh, Min Sun Kim, Yuno Do

**Affiliations:** 1Department of Biological Sciences, Kongju National University, Gongju 32588, Republic of Korea; jelee00@smail.kongju.ac.kr (J.-E.L.); jintall123@naver.com (Y.J.P.); 2Aquatic Disease Control Division, National Fishery Products Quality Management Service (NFQS), Busan 49111, Republic of Korea; mgkwon@korea.kr (M.-G.K.); oskal@korea.kr (Y.-K.O.)

## References

There were errors in the References section of the original publication [1]. Reference [21] had incorrect authors’ names and a error in the title; Reference [71] listed an incorrect set of co-authors. The authors state that the scientific conclusions are unaffected. This correction was approved by the Academic Editor. The original publication has also been updated.

21.Robinson, C.W.; McNulty, S.A.; Titus, V.R. No safe space: Prevalence and distribution of Batrachochytrium dendrobatidis in amphibians in a highly-protected landscape. *Herpetol. Conserv. Biol.* **2018**, *13*, 373–382.71.Herath, J.; Sun, D.; Ellepola, G.; Subramaniam, K.; Meegaskumbura, M. Emerging threat of ranavirus: Prevalence, genetic diversity, and climatic drivers of *Ranavirus* (*Iridoviridae*) in ectothermic vertebrates of Asia. *Front. Vet. Sci.* **2023**, *10*, 1291872.
